# Unified Alignment of Protein-Protein Interaction Networks

**DOI:** 10.1038/s41598-017-01085-9

**Published:** 2017-04-19

**Authors:** Noël Malod-Dognin, Kristina Ban, Nataša Pržulj

**Affiliations:** 1 Department of Computer Science, University College London, WC1E 6BT London, UK; 2 Laboratory of Data Technologies, Faculty of Information Studies, 8000 Novo Mesto, Slovenia

## Abstract

Paralleling the increasing availability of protein-protein interaction (PPI) network data, several network alignment methods have been proposed. Network alignments have been used to uncover functionally conserved network parts and to transfer annotations. However, due to the computational intractability of the network alignment problem, aligners are heuristics providing divergent solutions and no consensus exists on a gold standard, or which scoring scheme should be used to evaluate them. We comprehensively evaluate the alignment scoring schemes and global network aligners on large scale PPI data and observe that three methods, HUBALIGN, L-GRAAL and NATALIE, regularly produce the most topologically and biologically coherent alignments. We study the collective behaviour of network aligners and observe that PPI networks are almost entirely aligned with a handful of aligners that we unify into a new tool, Ulign. Ulign enables complete alignment of two networks, which traditional global and local aligners fail to do. Also, multiple mappings of Ulign define biologically relevant soft clusterings of proteins in PPI networks, which may be used for refining the transfer of annotations across networks. Hence, PPI networks are already well investigated by current aligners, so to gain additional biological insights, a paradigm shift is needed. We propose such a shift come from aligning all available data types collectively rather than any particular data type in isolation from others.

## Introduction

Sequence alignment has revolutionized our understanding of life. By finding correspondences between the genomes of different species, it gave us insights into function and phylogeny. However, genome is the blueprint of a cell: genes are transcribed into RNAs that are translated into proteins, and proteins interact with each other to perform biological functions. Thus, deciphering the connectivity patterns (also called topology) of protein-protein interactions (PPIs) is fundamental to understanding the functioning of the cell^1^. These interactions, which are increasingly available thanks to high throughput capturing methods such as yeast two-hybrid^2, 3^ and affinity purification coupled to mass spectrometry^4^, are commonly modelled as PPI networks, where nodes represent proteins and edges connect proteins that can interact. A large number of studies focuses on determining the commonalities and transferring annotation between PPI networks of different species, which is often done by global network alignments^5–10^. Globally aligning the PPI networks of two species means finding a one-to-one correspondence between the proteins of the two networks (also called an *alignment*) that highlights functionally and evolutionary conserved regions of the PPI networks. Global network alignments have uncovered valuable information, including evolutionary conserved pathways, protein complexes and functional orthologs^11–13^.

Unfortunately, network alignment is computationally intractable, due to NP-completeness of the underlying sub-graph isomorphism problem^14^ and thus, during the last decade, several heuristics (i.e., approximate aligners) have been proposed^12, 15–32^. These heuristics frequently combine two sources of information to guide their node mapping processes. On one hand, most use sequence similarity to measure the homology relationships between the proteins, as proteins having high sequence similarity are likely to have similar molecular functions (e.g., enzymatic activities)^33, 34^. On the other hand, network aligners are also using topological similarities (i.e., similarities between the wiring patterns around the proteins in the PPI networks), which provide information complementary to sequence about protein functions: proteins with similar patterns of interactions often have similar functions and the wiring patterns are conserved across species^35^. Also, to physically interact, proteins must be located in the same cellular component. Interacting proteins are also likely to belong to a common biological pathway. In general, network aligners combine sequence similarity between proteins (from BLAST^36^ sequence alignments) with a measure of similarity between their wiring patterns in the PPI networks (e.g., node degrees (numbers of neighbours of nodes in the network) for HUBALIGN^27^, spectral signatures for GHOST^22^, or graphlet degrees (number of small sub-graphs)^37, 38^ for L-GRAAL^29^) through a balancing parameter, so that the alignment can favour using sequence similarity, or topological similarity. Also, they use different algorithms for finding high-scoring alignments. Because of these differences, aligners provide different answers to the global network alignment problem. Global network aligners have been extensively reviewed and compared (e.g., refs 39–41), but no method has become a gold standard. In particular, the following key questions remain unanswered.

## Aligner and scoring selection

Due to a variety of heuristic aligners, choosing an appropriate network aligner is a difficult task, which is made harder by the large number of alignment quality measures that are available. Despite the wealth of review articles^39–41^, no consensus exists on which aligners should be used for which data, or which alignment scoring schemes should be used to evaluate them.

## Coverage and collective behaviour

The main limitation of global aligners is the coverage of their alignments. When aligning a small network to a large one, many proteins of the larger network are left unaligned, and no information can be gained for the unaligned proteins. To overcome this limitation, we need to investigate the collective behaviour of all aligners. If different network aligners consistently align the same regions of the PPI networks, then novel alignment algorithms are needed for extracting novel knowledge from the yet unexplored regions of the PPI networks. In contrast, if the PPI networks are well covered by the existing aligners, in the sense that entire PPI networks are aligned by the union of aligners, then we may have reached an upper limit on the amount of biological knowledge that can be extracted from PPI networks by aligning them and hence different approaches may be needed to uncover new biological knowledge about the cell.

## Topology versus sequence

Aligners combine sequence similarity with connectivity similarity to guide their alignment processes in order to produce more biologically relevant alignments than by using only sequences. However, the effect of the parameter that balances the amount of topological and sequence information that is used to guide the alignment processes is rarely reported. Hence, we have no clear guidance on how to set-up the parameters of the network aligners to produce the most biologically relevant alignments.

To answer these questions, we analyse the performance of the state-of-the-art global network aligners^20, 24–27, 29, 31, 32^ on the eight largest PPI networks from BioGRID^42^ (see Materials and methods). We assess the agreements between nine popular alignment scoring measures by using their Pearson’s correlations over thousands of alignments. These correlations show that all measures of topological quality are captured by the S^3^ score^26^, that all measures of biological quality are captured by the percentage of aligned proteins that share KEGG pathway annotations^43^, and that topological and biological scores largely disagree on recommending the best alignments. We present a comprehensive comparison of network aligners and find that HUBALIGN, L-GRAAL and NATALIE are the best performing, as they regularly produce the most topologically and biologically coherent alignments. Furthermore, we initiate the exploration of the collective behaviour of the union of network aligners and observe that their agreements are very low. However, when using all aligners together, the whole protein interaction mapping space is well covered. Thus, we propose a new tool, Ulign, that unify these global alignments. The multiple mappings of Ulign define biologically relevant soft clusterings of proteins in PPI networks, which can be used to transfer annotations across all proteins in PPI networks. This suggest that even if the individual performances of network aligners can be improved, PPI networks have already been well investigated by network aligners. Hence, to gain more knowledge on the investigated species, we propose that all data sources should be collectively aligned, possibly in the spirit of the recent data-integration methods where genes were clustered based on multiple data types^44–46^. Finally, we initiate investigation of the contribution of topological information to producing biologically relevant alignments.

## Materials and Methods

### Datasets

We use the PPI networks of 8 organisms with the largest and the most complete sets of physical protein-protein interactions from BioGRID (v3.2.101, June 2013)^42^: *Homo sapiens*, *Saccharomyces cerevisiae*, *Drosophila melanogaster*, *Arabidopsis thaliana*, *Mus musculus*, *Caenorhabditis elegans*, *Schizosaccharomyces pombe* and *Rattus norvegicus*. Both direct interactions (i.e., pairwise bindings captured by methods such as yeast-two-hybrid) and co-complex ones (capturing presence of proteins in stable complexes with methods such as affinity capture coupled with mass spectrometry) are included. We retrieve protein sequences from NCBI’s Entrez Gene database^47^ and compute their pairwise similarities using NCBI’s BLAST^36^. We also retrieve protein Gene Ontology (GO)^48^ annotations from NCBI’s Entrez Gene database and their Pathway annotations from KEGG^43^. Note that we only use experimentally validated GO annotations (i.e., we exclude the annotations predicted from computational analysis, such as sequence similarity).

Because the PPI networks of *Homo sapiens* and *Saccharomyces cerevisiae* are much larger and more complete than the other networks, we consider them separately, since it does not make sense to align a large and dense network with a small and sparse one. Thus, our study is based on 16 pairs of networks; the 15 pairs corresponding to all pairs of *Drosophila melanogaster*, *Arabidopsis thaliana*, *Mus musculus*, *Caenorhabditis elegans*, *Schizosaccharomyces pombe* and *Rattus norvegicus*; and the pair *Homo sapiens* and *Saccharomyces cerevisiae*. We do not consider synthetic networks generated from random graph models, as the behaviors of different network aligners on such data-sets are different than on real PPI networks^29, 31, 39^, which suggests that artificial networks do not properly reflect the real PPI network architectures.

### Network aligners

We briefly introduce the eight state-of-the-art pairwise network aligners that we study and describe the parameter settings that we used to run them.

**NATALIE** ^20^ is the first network aligner that formalizes network alignment as an integer program and that proposes an exact Lagrangian relaxation algorithm based on this formulation. However, to escape from NP-hardness, NATALIE only considers aligning proteins that are sequence similar. While this filtering largely reduces the search space, it also prevents NATALIE from discovering functionally related proteins that do not have homologous sequences.

**SPINAL** ^24^ uses a two-pass matching algorithm. The first pass consists of iteratively improving the estimated match confidence for each pair of nodes by taking into account the confidence of matching their neighbours that was computed in the previous iteration. After convergence of the first pass, the second pass consists of using a seed-and-extend algorithm to construct an alignment. Also, SPINAL has two distinctive modes, with Mode 1 performing the first pass and then simply performing a maximum-weight bipartite matching, whereas Mode 2 performs the two passes. On our datasets, only Mode 1 returned alignments for all PPI network pairs.

**PISWAP** ^25^ first identifies an optimal global alignment based purely on sequence data. Then, it uses the intuition that biologically conserved interactions can compensate for mapping proteins whose sequences are not particularly similar. In this way, the topology of the networks is taken into account and information is propagated from each vertex to its neighbours. The alignment itself is computed using a local “3-opt” heuristic, which is originally used for solving the travelling salesman problem^49^, and which consists of randomly swapping three edges when trying to improve the alignment score.

**MAGNA** ^26^ is the first genetic algorithm-based network aligner and aims at maximizing the edge conservation between the aligned networks. MAGNA directly optimizes edge-correctness, induced conserved sub-structure, or symmetric sub-structures scores, by using dedicated cross-over and fitness functions. We use MAGNA++^30^, which is an updated version of MAGNA that allows for parallel computations.

**HUBALIGN** ^27^ is based on the observation that proteins acting as hubs in the PPI networks are functionally and topologically more important, as their removal may disconnect functional parts of the interactomes^50^. HUBALIGN heuristically estimates likelihood of a protein to be a hub (which they call “importance” score) by iteratively peeling-off the nodes having the lowest degrees. Then, HUBALIGN uses a greedy seed-and-extend algorithm to align proteins based on the combination of their importance scores and sequence similarity.

**L-GRAAL** ^29^, unlike previous aligners that either do not take into account the mapped interactions or use naive interaction mapping scoring schemes, directly optimizes an objective function that takes into account both sequence-based protein conservation and topological, graphlet-based interaction conservation. L-GRAAL uncovers alignments by maximizing its objective function, by using an iterative double dynamic programming heuristic based on integer programming and Lagrangian relaxation.

**OPTNET** ^31^ uses a multi-objective memetic algorithm, coupling swap-based local search, mutation and crossover operations to create a population of alignments that optimize the conflicting goals of topological and sequence similarity. OPTNET uses the concept of Pareto dominance to explore the trade-off between the two objectives as it runs^51^.

**MODULEALIGN** ^32^, which is the most recent aligner, uses a hierarchical clustering of functionally related proteins to define its module-based homology scores between proteins. Then, it uses an iterative algorithm to find an alignment that maximizes a linear combination of its homology scores and of HUBALIGN’s importance scores.

We use the recommended settings of each network aligner. All methods except OPTNET have parameters that balance the amounts of sequence and topological information used to guide the alignments. For these methods, we sample these parameters from 0 to 1 in steps of 0.1 (i.e., for each pair of networks, for each of the methods, we generate 11 alignments). OPTNET is a genetic algorithm, which, by default, handles a population of 100 alignments, although it does not necessarily reach this upper-limit of 100 alignments. On average, it generates 85 alignments per network pair. Hence, in total, for our 16 pairs of networks, our study contains 2,770 alignments. We do not include ISORANK^15^, MI-GRAAL^12^, GHOST^22^, or NETAL^23^ in this study, as they fail to produce alignments for all of the pairs of networks that we consider. We also do not include DUALALIGNER^28^, as it requires GO annotations to produce alignments, while we use GO to assess the quality of the alignments.

### Scoring alignments

Over the past decade, numerous alignment scoring schemes have been proposed, measuring various aspects of alignment quality. However, there is no consensus on which scoring scheme should be used. We consider nine commonly used alignment scoring schemes, which we briefly present below (formulas are given in the Supplementary material).

#### Node coverage

The first measure of an alignment quality is the number of nodes/proteins that it maps between the networks. While global network aligners aim at aligning all the nodes of the smaller network to the larger one, they often fail to do so. *Node coverage* (NC) measures the number of mapped nodes normalized by the number of nodes in the smaller network.

#### Topological coherence

The topological similarity of the aligned regions of the networks was first assessed from the smaller networks point of view by *edge-correctness* ^12^ (EC), which is the percentage of edges from the smaller network that are aligned to some edges from the larger network. Although EC is an intuitive measure of an alignment quality, it only considers the smaller network. However, an alignment with large EC may map a sparse small network onto a dense region of a large network. Thus, the *induced conserved sub-structure score* ^22^ (ICS) considers the alignment from the larger networks point of view, by measuring the percentage of the edges from the aligned region of the larger network that are aligned to some edges from the smaller one. Hence, a large ICS allows a sparse region of the larger network to be mapped into a dense region of a small network. The *symmetric sub-structure score* ^26^ (S^3^) considers both networks by comparing the number of edges from the smaller network that are aligned to some edges from the larger network with: 1) the number of edges from the smaller network and 2) the number of edges in the sub-network of the larger network that is induced by its aligned nodes. Another popular measure of an alignment quality is the size of the largest aligned connected component of the two networks^12^, which measures how continuous the alignment is.

#### Biological coherence

KEGG pathway (KP) annotations^43^ are commonly used as a benchmark for protein functional similarity and two proteins are considered to be functionally similar if they participate in at least one common pathway. KP score is the number of aligned proteins that are functionally similar, divided by the smaller number of annotated proteins over the two networks. The same methodology is used with Gene Ontology (GO)^48^ annotations to measure the ability of an alignment to align proteins involved in similar biological processes (GO-BP score), having similar molecular functions (GO-MF score), or that are localized in the same cellular component (GO-CC score). In the Supplementary material, we additionally show that biological scores based on the semantic similarities between the GO annotations of the aligned proteins^20, 29^ strongly correlate with KP, GO-BP, GO-MF and GO-CC (see Supplementary Figure 1), which is why we do not include them in the main document.

## Results and Discussion

### Comparing alignment scoring methods

**Figure 1:**
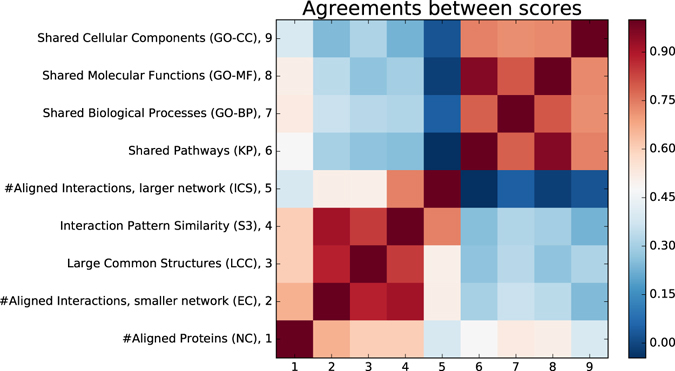
Relationships between alignment scores. The heat-map presents the agreements between the alignments scores, measured by their Pearson’s correlation coefficients (PCCs), which we computed over all alignments produced by all aligners. High PPC values (red) highlight the scores that are in good agreements, while low PCC values (blue) highlight the scores that have no agreements.

We assess the agreements of the above alignment scoring measures by finding Pearson’s correlation coefficients (PCCs)^52^ of these measures over all alignments produced by all aligners (2,770 alignments in total). The obtained PCCs, presented in Fig. 1, identify the following. The node coverage (NC) moderately correlates with all the other scores (average PCC of 0.52). This is expected, since global aligners aim at aligning all the proteins from the smaller network to the larger network, while producing topologically and biologically coherent alignments. In the group of topological quality scores, LCC, EC, S^3^ and ICS, the first three scores are all strongly correlated, with PCCs varying from 0.84 to 0.92, while ICS only strongly correlates with S^3^, with PCC of 0.74, and moderately correlates with EC and LCC (both with PCC of 0.51). Finally, all biological quality scores, KP, GO-BP, GO-MF and GO-CC are strongly correlated, with PCCs varying from 0.72 to 0.96.

From these correlations, we draw the following conclusions. First, all topological scores can be well subsumed by S^3^ score, which strongly correlates with EC, ICS and LCC. Also, all biological scores can be subsumed by KP score, which strongly correlates with GO-BP, GO-MF and GO-CC. Second, the low correlations (PCC of 0.22 on average) between the topological scores (EC, S^3^, ICS and LCC) and the biological scores (KP, GO-BP, GO-MF ad GO-CC) show that topological scores and biological scores disagree on which alignments are the best. This questions the relevance of considering network topology and topological scores in the context of biological network alignment, which we will further investigate in section “Relevance of topological information”.

### Comparing network aligners

**Figure 2:**
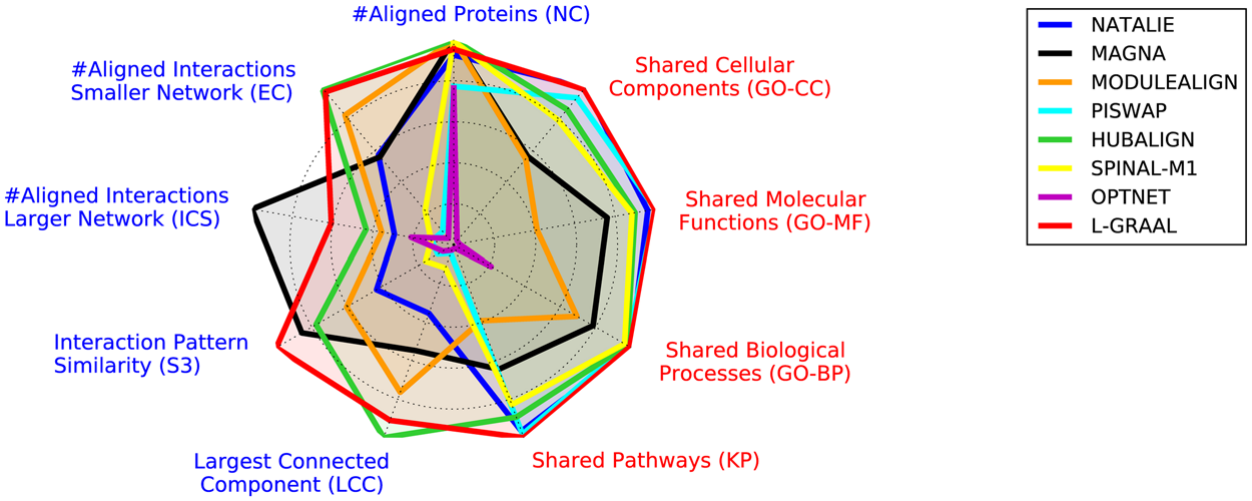
Comparing network aligners. Network aligners (coloured lines) are compared according to the best scores that they achieve when aligning the 16 pairs of networks (presented values are the averages of the 16 scores, normalized in [0,1] according to the best performers). Aligners are compared according to the nine scoring schemes: the five blue ones (on the left) are topology-based, while the four red ones (on the right) are biology-based (see definitions in Methods). The closer an aligner is to the border of the radar chart, the better it performs according to the corresponding score.

We compare the performances of aligners according to the nine alignment scores presented above. Recall that most of the aligners have a parameter in [0,1] that balances the amount of sequence and topological information that is used to guide the alignment process, which we sample from zero to one in steps of 0.1. For a given score, aligner and parameter setting, we compute the average of the scores of the alignments that the aligner produces by aligning the 16 pairs of PPI networks. We only report for a given scoring metric and a given aligner the highest average score that is obtained when the parameter varies from zero to one. The performances of the aligners are summarized in Fig. 2 and a simplified, rank-based comparison, is provided in Table 1. A detailed comparison of all aligners over all scores is presented in Supplementary Figure 2.

**Table 1:** Ranking network aligners.

Method	Coverage	Topological coherence				Biological coherence				Method rank	
	NC	EC	ICS	**S** ^**3**^	LCC	**KP**	GO-BP	GO-MF	GO-CC	**AVG (S** ^**3**^ **, KP)**	AVG (all)
L-GRAAL	5	2	2	**1**	2	**1**	2	2	2	**1**	2.11
HUBALIGN	1	1	3	**3**	1	**4**	4	4	4	**3.5**	2.78
NATALIE	6	4	5	**5**	5	**3**	1	3	1	**4**	3.67
MAGNA	1	5	1	**2**	4	**6**	6	6	6	**4**	4.11
PISWAP	7	7	8	**7**	7	**2**	3	1	3	**4.5**	5.00
MODULEALIGN	1	3	4	**4**	3	**7**	7	7	7	**5.5**	4.78
SPINAL	4	6	7	**6**	6	**5**	5	5	5	**5.5**	5.44
OPTNET	8	8	6	**8**	8	**8**	8	8	8	**8**	7.78

Network aligners (rows) are ranked according to the quality of their alignments: node coverage is measured with NC score (column 2), topological coherence is measured with EC, ICS, S^3^ and LCC scores (column 3 to 6) and biological coherence is measured with KP, GO-MB, GO-MF and GO-CC scores (column 7 to 10). Aligners are sorted according to the average of their ranks, which is computed over S^3^ and KP scores (column 11) and over all scores (last column).

While aligners map most of the smaller network’s nodes into the larger network’s ones (see Fig. 2), only three aligners, MAGNA, HUBALIGN and MODULEALIGN, succeed in entirely aligning the proteins from the smaller network to some proteins in the larger network, as evidenced by NC scores of 100%. They are closely followed by SPINAL (NC ≈ 99.4%), L-GRAAL (NC ≈ 95.2%) and NATALIE (NC ≈ 92.3%).

For the reasons presented above, we assess the ability of a network aligner to produce topologically coherent alignments by using S^3^ score. As presented in Fig. 2, L-GRAAL, HUBALIGN and MAGNA produce the alignments that best map topologically similar regions between the networks, with average S^3^ score of 29.7% for L-GRAAL, 25.4% for MAGNA and 23.0% for HUBALIGN. Because of their strong correlations with S^3^ score, comparisons based on EC, ICS and LCC also tend to identify these aligners as topologically the best performing (see Table 1); i.e., these aligners best map the interactions from the smaller network onto the larger one (EC score), they best map the interaction from the larger network onto the smaller one (ICS score) and they uncover the most continuous alignments (LCC score).

Similarly, for the reasons presented above, we assess the ability of network aligners to produce biologically relevant alignments by using KEGG pathway annotations. L-GRAAL, PISWAP and NATALIE best map proteins that are involved in similar pathways, with average KP score of 47.0% for L-GRAAL, 46.2% for PISWAP and 45.4% for NATALIE. Because of their high correlations with KP score, evaluations based on GO annotations also identify the same three aligners as top ranking (see Table 1); i.e., these methods best map proteins that are involved in similar biological processes (GO-BP score), that have similar molecular functions (GO-MF score) and that are located in the same cellular compartments (GO-CC score).

To identify the best performing aligners according to all scores, we globally rank the aligners using the average of their ranks over all scores. As presented in Table 1, L-GRAAL (average rank of 2.11), HUBALIGN (average rank of 2.78) and NATALIE (average rank of 3.67) regularly produce the most topologically and biologically coherent alignments. Also, we used the average of the ranks over S^3^ and KP scores and obtained the same ranking, which again confirms that all scores can be subsumed by S^3^ and KP.

Finally, we recall that the stated goal of network alignment is to produce alignments that simultaneously exhibit topological and biological coherence, which is not captured in our previous experiment (in which, for a given aligner, the alignments that maximize topological quality may not be the ones that maximize biological quality). To assess this ability, we follow the approach of Meng *et al*.^53^ and for a given alignment, we define the trade-off between its biological and topological quality as the geometric mean between its S^3^ and KP scores. Supplementary Figure 3 presents for each aligner the best trade-off that it achieves (average over all 16 pairs of networks) when parameter alpha (that balances the topological and sequence information that guide the alignment processes) varies in [0, 1] in steps of 0.1. Overall, L-GRAAL achieves the best trade-off (25.4%), followed by HUBALIGN (23.2%), MAGNA (19.5%) and NATALIE (19.3%).

### Union of aligners

#### Concurrent usage

Recall that global network aligners aim at aligning all proteins from the smaller network onto the larger one and that they leave many proteins from the larger network unaligned. We are interested in determining whether several network aligners can be used concurrently to align most of the proteins of the larger networks as well, or whether all aligners map the same regions of the larger networks. To investigate this, we define the *node mapping agreement* between two alignments, *i* and *j*, of the same network pair as follows. On the larger network, let *S*_*i*_ be the proteins that are mapped in alignment *i*, and *S*_*j*_ the ones that are mapped in alignment *j*. The agreement of the two alignments is the number of proteins of the larger network that is in the intersection of the two alignments, which we normalise in [0,1] by the number of proteins in the smaller alignment as: $agreement(i,j)=|{S}_{i}\cap {S}_{j}|/\,\min (|{S}_{i}|,|{S}_{j}|)$.

**Figure 3:**
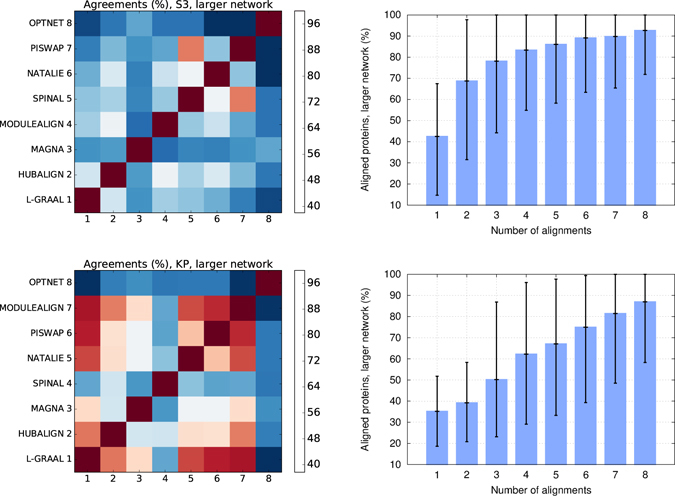
Agreements across aligners. We first consider the alignments that have the maximum topological similarity (S^3^). In the top-left panel, we report for each pair of aligners the average overlap between the proteins that they map on the larger networks, described by their node mapping agreement score. In the top-right panel, we report the average percentage of proteins from the larger networks that are aligned to some proteins from the smaller networks when considering simultaneously *k* different alignments (e.g., from *k* different aligners), with *k* = 0 (e.g., no alignment) to 8 (when considering one alignment form each of the eight aligners). Alignments are successively added according to the performance of the aligners for the considered score (best aligner first); i.e., aligners are considered in the following order: L-GRAAL, MAGNA, HUBALIGN, MODULEALIGN, NATALIE, SPINAL, PISWAP and OPTNET. The bottom-panels show analogous but when considering the alignments that have maximum biological similarity (KP) and aligners are considered in the following order: L-GRAAL, PISWAP, NATALIE, HUBALIGN, SPINAL, MAGNA, MODULALIGN and OPTNET (for the reasons analogous to above).

For each aligner, we first pick the alignment for which the topological similarity, as measured by S^3^ score, is the largest (over the alignments produced by the aligner with the value of the balancing parameter in [0,1] in increments of 0.1). For these alignments on our 16 pairs of networks, the node mapping agreement between any two aligners is 57.87% on average (from 38.05% between PISWAP and OPTNET, to 85.04% between PISWAP and SPINAL, see the top-left panel of Fig. 3), meaning that these alignments map proteins of the smaller networks to different proteins from the larger networks. Interestingly, these low node mapping agreements allow the network aligners to jointly cover all proteins from the larger network: the union of the alignments from each aligner with the largest S^3^ (i.e., the eight alignments for a given pair of PPI networks, one for each of the eight aligners) maps 92.66% of the proteins of the larger network on average. This is in sharp contrast with mapping only 42.53% of the nodes of the larger network when considering only one alignment (top-right panel of Fig. 3). Similarly, we consider the alignments with the maximum biological coherence (as measured by KP score) and find the node mapping agreements between the aligners to be 65.79% on average (from 37.68% between L-GRAAL and OPTNET, to 94.40% between L-GRAAL and MODULEALIGN, see the bottom-left panel of Fig. 3). The union of the alignments with maximum KP score from each aligner allows for mapping of 86.98% of the nodes of the larger network on average (bottom-right panel of Fig. 3). This is lower than when S^3^ is maximized, due to the larger node mapping agreements when we maximize KP score. These results show that due to the differences in the objective functions and the alignment search strategies, taken together, network aligners map most of the proteins from the larger networks as well.

Alternatively, we also measure the agreement between two alignments, *i* and *j*, of the same network pair as the percentage of identical protein mappings (with respect to the smaller alignment). For the above presented alignments that maximize topological coherence (S^3^ score), there is only 2.42% of identical protein mappings between two aligners on average (from 0.730% between L-GRAAL and OPTNET to 41.92% between SPINAL and PISWAP, see Supplementary Figure 4, left). For the above presented alignments that maximize biological coherence (KP score), there is about 24.73% of identical protein mappings between two aligners on average (from 0.97% between L-GRAAL and OPTNET to 75.37% between L-GRAAL and PISWAP, see Supplementary Figure 4, right). These results suggest that global aligners tend to produce good (with large S^3^ or KP scores) yet disjoint alignments.

#### Unifying global network alignments

The complementarity of network aligners, whose concurrent usage allows for finding a mapping for most of the larger (and also smaller) network’s proteins, allows us to fully align both the smaller and the larger network by taking the union of the alignments. Hence, we provide “Ulign”, a software for unifying alignments from different network aligners. Note that the resulting alignments are not one-to-one node mappings any more (a node from the smaller network can be aligned to several nodes from the larger network) and thus cannot be evaluated using the traditional network alignment quality measures.

Ulign’s alignments may look similar to local network alignments, since both are many-to-many mappings. However, local aligners focus on the most conserved regions of the networks, and thus return very small alignments. Although local aligners are not designed to align large networks (AlignNemo^54^, AlignMCL^55^ and LocalAli^56^ all encounter memory issues on our datasets), we were able to use AlignNemo to align our smallest pair of networks, *R. norvegicus* PPI network (1,657 nodes and 2,330 edges) and *S. pombe* PPI network (1,911 nodes and 4,711 edges). Between these two networks, AlignNemo produced 209 local alignments, each aligning 9.3 proteins from each network on average. All-together, these alignments map 42% of the proteins from the smaller network to 41% of the protein from the larger one. In the same vein, global aligners can align at most 1657/1911 = 86.7% of the proteins from the larger network. In contrast, by using only eight global alignments, Ulign succeeds in mapping all proteins between the two networks. Thus, Ulign can be seen as a local aligner that fully aligns the networks.

Furthermore, Ulign can also be used for annotation transfer accross PPI networks. When Uligning the PPI networks of yeast *S. cerevisiae* (5,831 nodes and 77,149 edges) and human (13,276 nodes, 110,528), the multiple mappings from the alignments from each of the eight aligners that maximize biological KP score) imply soft (overlapping) clustering of yeast’s proteins which are grouped together if they are mapped to the same human protein. To assess if these clusters are biologically relevant, we consider the clusters having at least two annotated proteins (using GO biological process annotations) and compute their enrichment in GO terms using sampling without replacement and Benjamini-Hochberg correction. We find that these clusters are highly biologically relevant, since ~99% of these clusters have at least one significantly enriched GO biological process annotation (with enrichment p-values ≤ 5% after correction). Furthermore, the biological consistency of the mapping by Ulign from these clusters towards human proteins is also very high; in 46% of the cases in which the mapped human protein is also annotated, at least one annotation from the yeast cluster (i.e., from the union of the annotations of all proteins in the cluster) can be found within the annotations of the corresponding mapped human protein. The same is observed when using Kegg pathway, GO molecular function and GO cellular component annotations (see Supplementary Table 1). These results suggest that Ulign’s clusters are biologically relevant and may be used to transfer annotations.

As we can now fully align PPI networks by using only a handful of aligners concurrently, we can find correspondences and thus transfer knowledge for any protein in a PPI network. This calls for an evaluation of the entire PPI network alignment paradigm. Indeed, global aligners can still be improved by defining novel protein similarity measures and more advanced alignment algorithms, but these improvement will be limited in the sense that they only use the PPI data. Recall that PPI networks are particularly incomplete; for example, our human PPI network has some interactions for 13,276 proteins, with most of them still being unknown. Other systems level omics data sets, such as gene co-expression^57^ and genetic interaction networks^58^, could provide additional and complementary information to the PPI data. Hence, we believe that a leap forward in biology may lie in concurrent alignment of different molecular data types, rather than from aligning any data type in isolation from others, such as aligning PPI networks to PPI networks, or genetic sequences to sequences, as was done thus far. The power of fusion of different data types has already been demonstrated in other biological and medical applications^44, 45, 59, 60^ and it is time to apply it to the alignment problem as well^61^.

### Relevance of topological information

Our analysis of various alignment scoring schemes showed that the topological scores are not in agreement with biological coherence of alignments. Going further, we investigate if using topological information for producing the alignment is relevant in the context of producing alignments that are more biologically relevant than the alignments obtained when using sequence information alone. To this aim, we report for the four best aligners (L-GRAAL, HUBALIGN, NATALIE and MAGNA) the average quality of their alignments when their alignment processes are guided from topological information to sequence information only. The quality of the alignments are measured using one topological scores (S^3^), one biological annotation based scores (KP) and the trade-off score between topological and biological quality.

**Figure 4:**
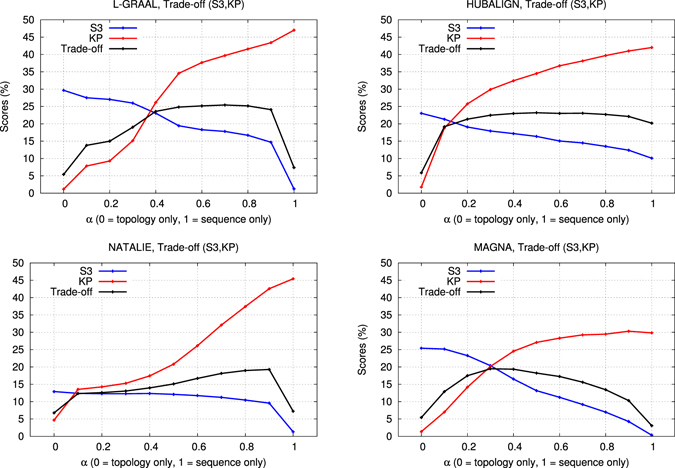
Effects of using topology and sequence homology. For L-GRAAL (top-left panel), HUBALIGN (top-right panel), NATALIE (bottom-left panel) and MAGNA (bottom-right panel), we report the average quality of the alignments that they produce on our 16 PPI network pairs, when aligning PPI networks by using topological information only (*α* = 0, on the left on the x-axis), sequence similarity only (*α* = 1, on the right on the x-axis) and their combinations (all *α* in between in increments of 0.1). The qualities of the alignments are measured with topological score S^3^ (blue curves), biological score KP (red curves) and the trade-off score between biological and topological quality (black curves).

When the alignment processes are guided by topological information only, aligners produce alignments having the highest topological coherence and the lowest biological coherence (see Fig. 4). In contrast, when aligners are guided by sequence information only, they produce alignments having the highest biological coherence and the lowest topological coherence. As a minor remark, this explains why in Fig. 2 network aligners have larger variations in their topological scores than in their biological scores; the most topologically coherent alignments are obtained using topological information only, which can be very different across aligners, while the most biologically coherent alignments are all obtained from sequence information. More importantly, this result shows that network aligners do not succeed in using topological information (even in combination with sequence information) to produce alignments that are biologically more relevant than the alignments based solely on sequence information.

This may be a result of the existing bias of biological annotations towards sequence similarity. KEGG pathways are reconstructed from homology relationships that are based on multiple sequence alignments. Similarly, while we only considered the GO annotations that are experimentally validated, we recall that such experiments are costly and are mostly done to validate predicted functions, which in most cases are sequence based. This may imply that topology based alignments are biologically relevant, but that using the existing biological annotations to measure their relevance is ill-suited. On the other hand, we recall that limitations in capturing technologies, promiscuous non-functional interactions and sample biases make PPI networks noisy, which may blur the biological signal that they contain^62, 63^. Because of computational hardness, topological similarity between proteins in PPI networks is heuristically investigated, and current methodologies used by network aligners may not be suited. However, a recent study suggests that the problem comes from methodological limitations in how network aligners combine topological and sequence-based similarity^64^. Aligners frequently use simple linear combinations and these naive strategies may not be able to properly extract biologically relevant association between the proteins. In contrast, Gligorijevic *et al*.^64^ successfully used a data integration technique called non-negative matrix tri-factorization to combine topological and sequence-based similarities in a more principled way that produced novel pairwise scores between the proteins that are in better agreements with biological annotations (GO biological processes and GO molecular functions) than the sequence similarities can produce. Using these integrated similarities between proteins, they proposed a multiple network aligner (Fuse) which produces alignments that are biologically more relevant than the alignments obtained when using sequence similarities only. Thus, a key problem that needs to be solved in global network alignment is the definition of a proper framework for combining topology and sequence information to produce more biologically relevant network alignments. Data integration techniques, such as the one used in Fuse, show promise in this respect.

### Concluding Remarks

We present an extensive comparison of network aligners and of the scoring schemes used to assess the quality of the produced alignments. On the largest PPI networks from BioGRID, we show that three methods, HUBALIGN, L-GRAAL and NATALIE, regularly produce the most connectivity coherent and the most biologically relevant alignments. When using all aligners together, we observe that eight different aligners are enough to map almost all proteins between both PPI networks, meaning that even if the individual performances of particular network aligners could be improved, PPI networks can already be well investigated by the existing network aligners. We provide a tool, “Ulign”, that does this. As using eight aligners concurrently is enough to find mappings and transfer annotations across all proteins of the PPI networks, this calls for a paradigm shift in alignment-based research in biology. In particular, we may have reached the upper limit in biological information that could be extracted by aligning PPI networks, or any other molecular data type in isolation from other molecular data types. A leap forward is likely to lie in holistically aligning all types of molecular data, including sequences, protein interactions, co-expression data, genetic interactions, metabolic reactions, epigenetic data etc. Coupling machine learning with network analytics may be a way forward in that direction.

## Electronic supplementary material

Supplementary material
