# Supplementary material for: Unified Alignment of Protein-Protein Interaction Networks
Source: Sci Rep. 2017 Apr 19;7:953. doi: 10.1038/s41598-017-01085-9 (PMC5430463; doi:10.1038/s41598-017-01085-9)
Supplement: Supplementary file 1 — Supplementary material [file 41598_2017_1085_MOESM1_ESM.pdf]

Unified Alignment of Protein-Protein Interaction Networks: Supplementary Material

Noël Malod-Dognin[1], Kristina Ban[2], and Nataša Pržulj[1,*]

[1]Department of Computer Science, University College London, WC1E 6BT London, United Kingdom
[2]Laboratory of Data Technologies, Faculty of Information Studies, 8000 Novo Mesto, Slovenia
[*]natasha@cs.ucl.ac.uk

Alignment scores

We use the following definitions and notations throughout the rest of this section. The PPIs of two species are represented by two PPI networks, $N_1 = (V_1, E_1)$ and $N_2 = (V_2, E_2)$, where nodes in $V_i (i = 1,2)$ represent proteins and two proteins are connected by an edge in $E_i (i = 1,2)$ if they interact. We assume that N_1 is smaller than or equal to N_2, i.e., that $|V_1| \leq |V_2|$.

An alignment, a, between the two PPI networks defines a common sub-network, $A = (V_a, E_a)$, whose nodes in V_a are the aligned nodes $u \leftrightarrow w$, $u \in V_1$, $w \in V_2$, and whose edges in E_a are the aligned edges $(u,v) \leftrightarrow (w,x)$, $(u,v) \in E_1$, $(w,x) \in E_2$. Finally, the aligned region on the larger graph (i.e., the sub-network of N_2 that is induced by its aligned nodes in V_a) is denoted by $N_2[V_a]$, and the edge set of this sub-graph is denoted by $E_{N_2[V_a]}$.

We now present the topological and biological scores that we use in our study to assess the quality of alignments.

Node coverage. A frequently overlooked measure of the quality of an alignment is how many nodes/proteins it maps between networks. In effect, while global network aligners aim at aligning all nodes of the smaller network to the nodes of the larger one, they often fail to do so. This is measured by *node coverage* (NC), where the number of mapped nodes is normalized in [0,1] according to the number of nodes in the smaller network:

$$NC(a) = \frac{|V_a|}{|V_1|} \times 100\%. \tag{1}$$

Edge correctness[1]. A popular measure of an alignment quality is the percentage of the interactions (edges) from the smaller network that are aligned to some edges from the larger one. This is measured by *edge-correctness* (EC):

$$EC(a) = \frac{|E_a|}{|E_1|} \times 100\%. \tag{2}$$

Induced conserved sub-structure score[2]. Although EC is an intuitive measure of an alignment quality, it only considers the smaller network. An alignment with large EC may map a sparse small network onto a dense region of the large network. Thus, the *induced conserved sub-structure score* (ICS) considers the alignment from the larger network's point of view, by measuring the percentage of the interactions from the aligned region of the larger network that are aligned to some interaction from the smaller one. It is defined as:

$$ICS(a) = \frac{|E_a|}{|E_{N_2[V_a]}|} \times 100\%. \tag{3}$$

Symmetric sub-structure score[3]. While a large EC allows a sparse small network to be mapped onto a dense region of the larger network, a large ICS allows a sparse region of the larger network to be mapped onto a dense region of the smaller network. The *symmetric sub-structure score* (S^3) considers both networks by comparing the number of aligned edges to the number of edges of the smaller network and to the number of edges in the aligned region of the larger network. It is defined as:

$$S^3(a) = \frac{|E_a|}{|E_1| + |E_{N_2[V_a]}| - |E_a|} \times 100\%. \tag{4}$$

Size of the largest common connected component[1]. Another popular measure of an alignment quality is the size of the largest connected component (LCC) shared by the two graphs (i.e., the largest connected component of that is found in A). A larger LCC implies that the alignment contains a larger amount of shared continuous structure between the two networks.

Biological annotation based scores. The above presented measures focus on the topological quality of an alignment and they give no information about the functional similarity of the aligned proteins. First, we use Kegg pathway annotations[4] as a benchmark for protein functions and we consider two proteins to be functionally similar if they participate in at least one common pathway. Then, we measure the ability of an alignment to align pathways as the number of aligned proteins that are functionally similar divided by the smaller number of annotated proteins over the two networks (KP). We use the same methodology with Gene Ontology (GO)[5] annotations to measure the ability of an alignment to align proteins involved in similar biological processes (GO-BP), having similar molecular functions (GO-MF), or that are localized in the same cellular component (GO-CC).

GO's semantic similarities of aligned proteins[6,7]. Another popular measure of functional similarity between two proteins is the semantic similarity of their GO annotations. There exist two types of semantic similarities. Node-based semantic similarity defines the information content of a term as a function of its frequency of appearance in the annotated dataset and measures the similarity between two terms according to their most informative ancestor in the ontology[8–10]. Edge-based semantic similarity only uses the ontology's directed acyclic graph and measures the similarity between two terms based on the shortest path between them, or based on the depth in the ontology of their common ancestors[11]. We compute the semantic similarity between the annotations of two proteins using Resnik (node-based) semantic similarity[10] with best-match average mixing strategy, because it achieves higher and more consistent correlations with molecular interaction data than other approaches[12–14]. Then, we measure the biological quality of an entire alignment as the sum of semantic similarities of the aligned proteins, divided by the smaller number of annotated proteins over the two networks. We consider separately the average semantic similarity of biological process annotations (SS-BP), the average semantic similarity of molecular function annotations (SS-MF) and the average semantic similarity of cellular component annotations (SS-CC). Because of their large correlation with KP, GO-BP, GO-MF and GO-CC scores (see supplementary Figure 1), we do not report results on SS-BP, SS-MF and SS-CC scores in the main document.

References

1. Kuchaiev, O., Milenković, T., Memišević, V., Hayes, W. & Pržulj, N. Topological network alignment uncovers biological function and phylogeny. *Journal of The Royal Society Interface* **7**, 1341–1354 (2010).
2. Patro, R. & Kingsford, C. Global network alignment using multiscale spectral signatures. *Bioinformatics* **28**, 3105–3114 (2012).
3. Saraph, V. & Milenković, T. Magna: Maximizing accuracy in global network alignment. *Bioinformatics* **30**, 2931–2940 (2014).
4. Kanehisa, M. & Goto, S. Kegg: kyoto encyclopedia of genes and genomes. *Nucleic Acids Research* **28**, 27–30 (2000).
5. Ashburner, M., Ball, C. A., Blake, J. A. *et al.* Gene ontology: tool for the unification of biology. *Nature Genetics* **25**, 25–29 (2000).
6. El-Kebir, M., Heringa, J. & Klau, G. Lagrangian relaxation applied to sparse global network alignment. In Loog, M., Wessels, L., Reinders, M. & Ridder, D. (eds.) *Pattern Recognition in Bioinformatics*, vol. 7036 of *Lecture Notes in Computer Science*, 225–236 (Springer Berlin Heidelberg, 2011).
7. Malod-Dognin, N. & Pržulj, N. L-GRAAL: Lagrangian graphlet based network alignment. *Bioinformatics* **31**, 2182–2189 (2015).
8. Lin, D. An information-theoretic definition of similarity. In *Proceedings of the Fifteenth International Conference on Machine Learning*, 296–304 (Morgan Kaufmann Publishers Inc., 1998).
9. Resnik, P. Using information content to evaluate semantic similarity in a taxonomy. In *Proceedings of the 14th International Joint Conference on Artificial Intelligence*, 448–453 (1995).
10. Resnik, P. Semantic similarity in a taxonomy: An information-based measure and its application to problems of ambiguity in natural language. *Journal of Artificial Intelligence Research* **11**, 95–130 (1999).
11. Cheng, J. *et al.* A knowledge-based clustering algorithm driven by gene ontology. *Journal of Biopharmaceutical Statistics* **14**, 687–700 (2004).
12. Sevilla, J. L. *et al.* Correlation between gene expression and go semantic similarity. *IEEE/ACM Transactions on Computational Biology and Bioinformatics* **2**, 330–338 (2005).

13. Pesquita, C., Faria, D., FalcÃ£o, A. O., Lord, P. & Couto, F. M. Semantic similarity in biomedical ontologies. *PLoS Computational Biology* **5** (2009).

14. Guzzi, P. H., Mina, M., Guerra, C. & Cannataro, M. Semantic similarity analysis of protein data: assessment with biological features and issues. *Briefings in Bioinformatics* **13**, 569–585 (2012).

Supplementary figures

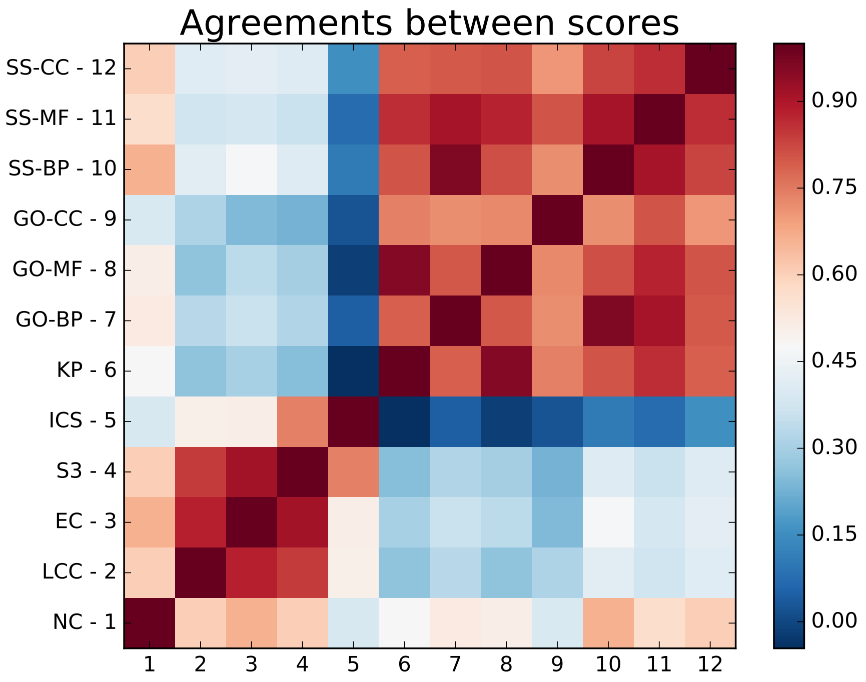

Supplementary Figure 1. Relationships between alignment scores. The heat-map presents the agreements between the alignments scores, measured by their Pearson's correlation coefficients (PCCs), which we computed over all alignments produced by all aligners. High PCC values (red) highlight the scores that are in good agreements, while low PCC values (blue) highlight the scores that have no agreements.

Annotations	% Enriched Clusters	% Validated Clusters
Kegg Pathways	60.3% (of 1,429)	67.9% (of 977)
GO-BP	99.2% (of 6,108)	46.4% (of 5,339)
GO-MF	98.0% (of 5,040)	59.9% (of 4,547)
GO-CC	70.9% (of 6,556)	71.1% (of 5,989)

Supplementary Table 1. Biological quality of Ulign's soft clustering. Ulign's mapping between yeast and human induces a soft (overlapping) clustering on yeast's proteins (grouping together the proteins from yeast that are mapped to the same protein in human). According to four different gene annotations (column 1), we report the percentages of these clusters that have at least one enriched annotation (with respect to the clusters having at least two annotated proteins, which is the number in parentheses). We also measure the percentage of these clusters that are "Valid" with respect to their mapped human proteins (column 3), which we define as the clusters having at least one annotation that is found within the annotations of their mapped human proteins (with respect to the number of these clusters that are mapped with an annotated protein in human).

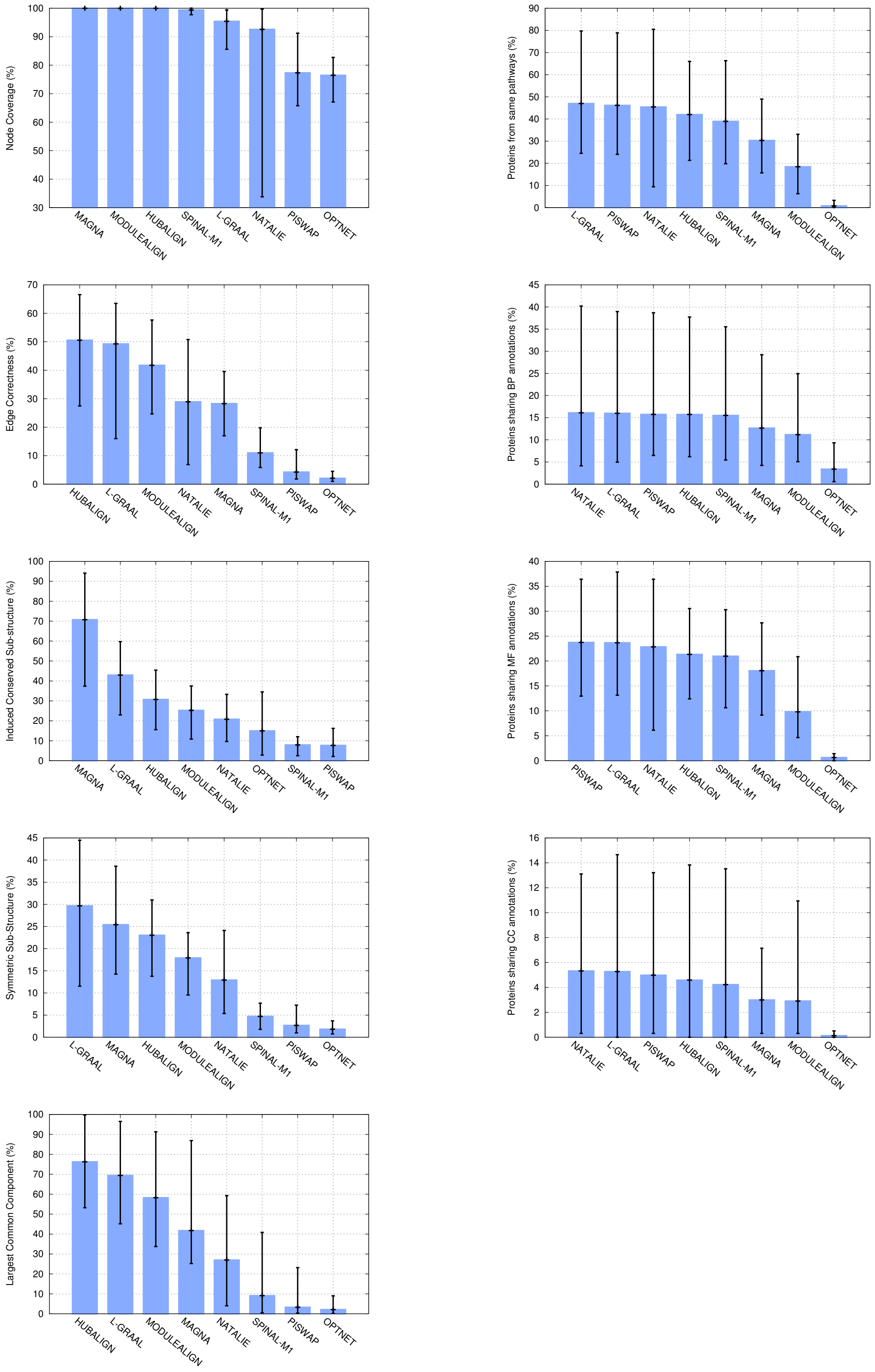

Supplementary Figure 2. Detailed performance comparisons. Network aligners (x-axis) are compared according to the topological and biological quality (y-axis) of their alignments. The error bars show the smallest, the average and the maximum of these scores over the 16 PPI network pairs, respectively. The left panels present the results for the topological scores (from top to bottom: NC, EC, ICS, S^3 and LCC) and the right panels present the results for the biological scores (from top to bottom: KP, GO-BP, GO-MF and GO-CC). In each panel, aligners are sorted from the best performing (on the left) to the worst performing method (on the right).

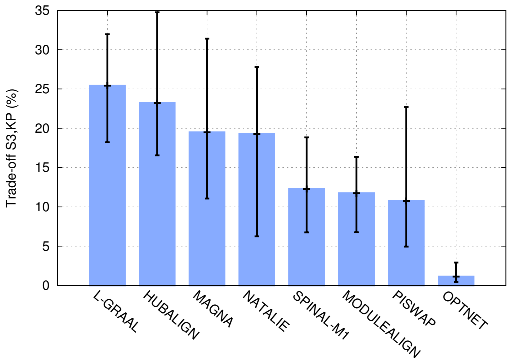

Supplementary Figure 3. Trade-off between topological and biological quality. Network aligners (x-axis) are compared according to the trade-off scores of their alignments (geometric mean of S^3 and KP scores, y-axis). The error bars show the smallest, average and maximum of these scores over the 16 PPI network pairs, respectively. Aligners are sorted from the best performing (on the left) to the worst performing method (on the right).

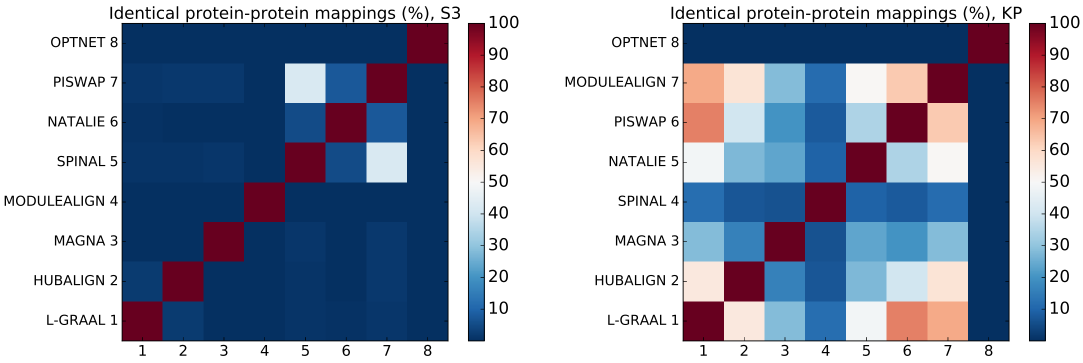

Supplementary Figure 4. Percentages of identical protein mappings between aligners. We first consider the alignments that have the maximum topological similarity (S^3). In the left panel, we report for each pair of aligners the average percentage of identical protein-protein mappings found in their alignments, described by their node mapping agreement score. The right panel shows the same, but when considering the alignments that have maximum biological similarity (KP).
